# Internal Hernia of the Greater Omentum: Cadaveric Findings of a Previously Unreported Variant

**DOI:** 10.7759/cureus.1294

**Published:** 2017-05-30

**Authors:** Andrea Andrea, Vy Tran, Cameron K Schmidt, Christian Fisahn, Joe Iwanaga, Rod J Oskouian, R. Shane Tubbs

**Affiliations:** 1 Seattle Science Foundation; 2 Clinical Anatomy, Seattle Science Foundation; 3 Orthopedic Surgery, Swedish Neuroscience Institute; 4 Neurosurgery, Complex Spine, Swedish Neuroscience Institute; 5 Neurosurgery, Seattle Science Foundation

**Keywords:** transomental hernia, greater omentum, anatomy, internal hernia, intestinal obstruction

## Abstract

Transomental hernias (TOHs) are a rare finding, constituting a fraction of all intestinal hernias. Here, we report the cadaveric discovery of a spontaneous TOH involving the sigmoid colon in an 82-year-old female and discuss the relevant literature. To our knowledge, a TOH involving the sigmoid colon has not been previously reported.

## Introduction

An internal hernia is characterized by the protrusion of the viscera through a congenital or acquired peritoneal or mesenteric aperture within the peritoneal cavity [1-5]. Internal hernias are an uncommon cause of small bowel obstructions, accounting for fewer than six percent of the cases [1,4-6]. Representing only a fraction of all intestinal hernias, transomental hernias (TOHs) are exceedingly rare [1-5,7]. In exceptional cases, TOHs may occur spontaneously in the absence of congenital anomalies or traumatic abdominal history [3,7-8]. Here, we report the discovery of a spontaneous TOH involving the sigmoid colon, incidentally discovered during cadaveric dissection.

## Case presentation

**Figure 1:**
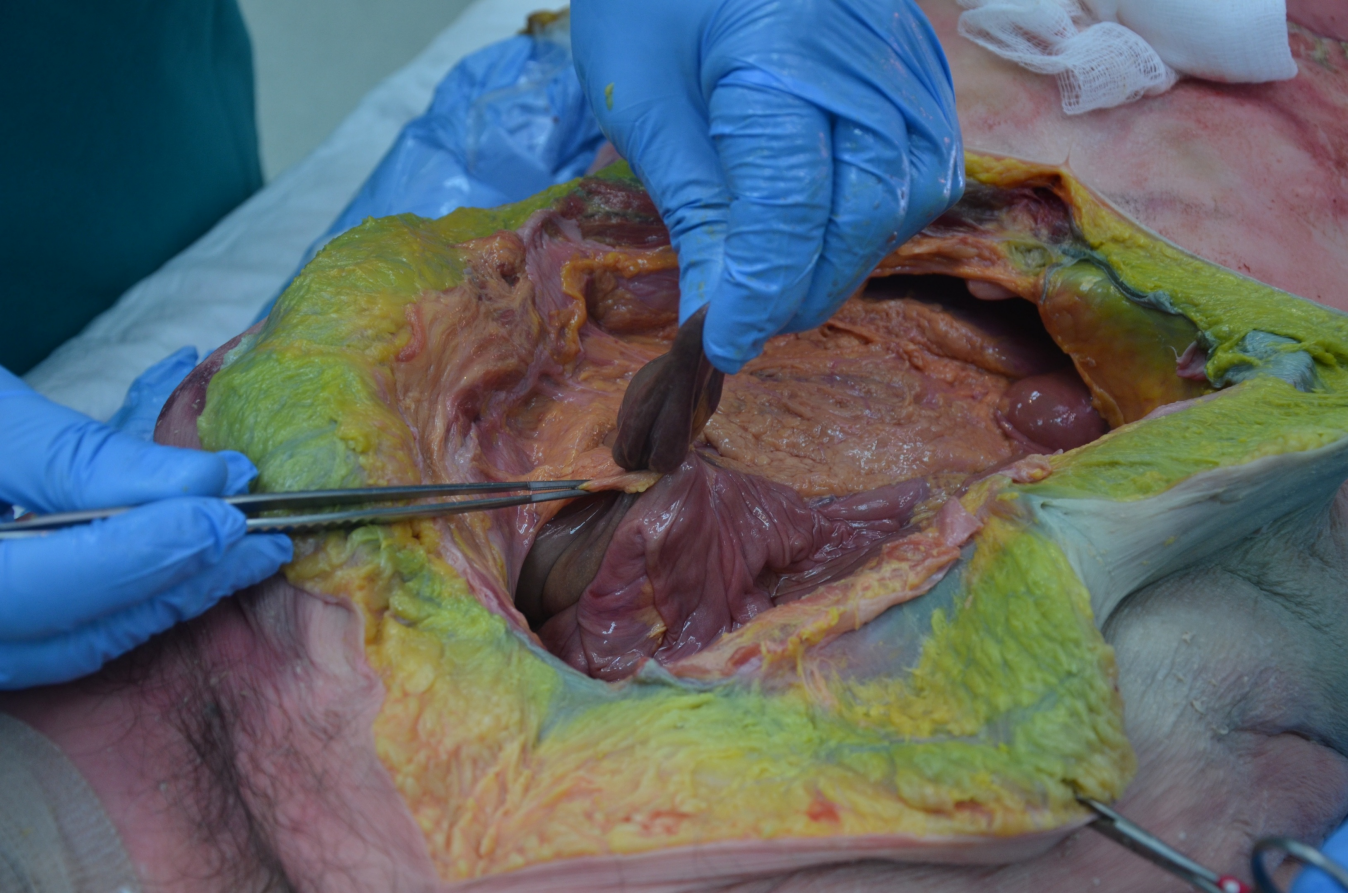
Intraabdominal view of the herniated sigmoid colon Intraabdominal view of the herniated sigmoid colon (held by left hand). Note the greater omentum (forceps) and narrow defect where the herniated colon has traversed.

During the routine dissection of the abdomen of a fresh adult female cadaver, aged 82 years at the time of death, an entrapped segment (approximately 5 cm in length) of the sigmoid colon was identified as seen in Figure 1.

The cause of death was pneumonia and there was no history of abdominal surgery. The herniated piece of sigmoid colon was strangulated but not necrotic (although the coloration was slightly darker than its proximal and distal non-trapped sigmoid colon segments) and traversed a small part of the free edge of the greater omentum. The defect in the greater omentum measured 1 cm in diameter. No masses or congenital malformations were found in the region of the herniation. The regional abdominal viscera were found to be within normal limits.

## Discussion

TOHs are exceptionally rare, commonly cited to account for roughly one percent to four percent of all internal hernias, although this may be an underestimate [2,4-6]. In their series, Blachar and Federle found TOHs to constitute 5.5% (three out of 54 cases) of internal hernias, while among the 49 internal hernia cases reviewed by Ghiassi et al., roughly 10% (five cases) were TOHs [4-5].

Preoperative diagnosis is made difficult by few specific clinical manifestations. Patients typically present with symptoms resembling acute abdominal obstruction, including acute onset of abdominal pain, nausea, vomiting, constipation, and abdomen distension [2-4,6-7]. TOHs have a number of characteristic computed tomography (CT) findings, including the whirl sign (characterized by a swirling and stretching of the mesentery), the beak sign (characterized by the triangular formation created by the loop of closely apposed small bowels inside the intraperitoneal hernial ring), clusters of dilated small bowel loops (air or fluid-filled), intestinal infarction signs, and displacement of the ascending colon and cecum medially and posteriorly [1-5,7-8]. However, similar to its clinical presentation, the radiographic presentation of a TOH is non-specific and the condition is easily misdiagnosed [7-8]. In a series of 49 surgically diagnosed internal hernias, it was reported that only 16% of preoperative CT scans were considered suspicious for an internal hernia [4].

While TOHs occur in both children and adults, they are most often seen in patients over the age of 50 years [4]. The hernial defects in pediatric cases tend to be congenital in nature. In adulthood, defects are largely iatrogenic, arising from trauma secondary to surgery, inflammation, or blunt force trauma [3,7-8]. In rare cases, hernias occur spontaneously, presumably as a result of senile atrophy of the omentum. Lee and Lee report a case of spontaneous TOH in a 57-year-old male, initially presenting with abdominal pain and vomiting over the previous 24 hours [7]. At presentation, the patient was believed to be experiencing strangulation of the small bowel secondary to an internal hernia. Not until surgery was a TOH diagnosed. With no previous history of abdominal trauma or surgery, this case represented a spontaneous herniation. Based on age, the cadaveric case reported here most likely represents a senile atrophy of the greater omentum with spontaneous herniation.

Internal hernias harbor a high mortality rate, given the ease of gangrene development among other complications [6]. As such, early diagnosis and surgical intervention are central to the treatment of these cases. Reduction of the herniated intestinal elements is the primary goal in surgery [7]. If necrosis has occurred, this tissue must be excised before a repair of the defect is performed, in order to prevent further herniation.

## Conclusions

Representing roughly 1% to 10% of all internal hernias, spontaneous TOHs are exceedingly rare and generally difficult to diagnose without abdominal exploratory surgery. Here, we have reported the discovery of a spontaneous TOH involving the sigmoid colon and discussed the relevant literature surrounding internal hernias. To our knowledge, a TOH involving the sigmoid colon has not been previously reported.
